# Nicotine-Induced Expression of Low-Density Lipoprotein Receptor in Oral Epithelial Cells

**DOI:** 10.1371/journal.pone.0082563

**Published:** 2013-12-16

**Authors:** Satoshi Ito, Takahiro Gojoubori, Kou Tsunoda, Yoko Yamaguchi, Masatake Asano, Eiji Goke, Ryosuke Koshi, Naoyuki Sugano, Naoto Yoshinuma, Kazuo Komiyama, Koichi Ito

**Affiliations:** 1 Division of Applied Oral Sciences, Nihon University Graduate School of Dentistry, Tokyo, Japan; 2 Department of Biochemistry, Nihon University School of Dentistry, Tokyo, Japan; 3 Division of Functional Morphology, Nihon University School of Dentistry, Tokyo, Japan; 4 Department of Pathology, Nihon University School of Dentistry, Tokyo, Japan; 5 Division of Immunology and Pathobiology, Nihon University School of Dentistry, Tokyo, Japan; 6 Department of Periodontology, Nihon University School of Dentistry, Tokyo, Japan; 7 Division of Advanced Dental Treatment, Nihon University School of Dentistry, Tokyo, Japan; 8 Nihon University School of Dentistry, Tokyo, Japan; University of North Carolina at Chapel Hill, United States of America

## Abstract

**Background:**

Nicotine use is one of the most important risk factors for the development of cardiovascular and periodontal diseases. Numerous reports have suggested the possible contribution of disturbed lipid metabolism for the development of both disease groups. Despite these observations, little is known about the relationship between tobacco smoking and the development of these diseases. Our previous microarray data revealed that nicotine induced low-density lipoprotein receptor (LDLR) expression in oral epithelial cells (OECs). The aim of the present study was to confirm nicotine-mediated LDLR induction and to elucidate the signaling mechanisms leading to the augmented expression of LDLR in OECs.

**Methods and Results:**

LDLR and nicotinic acetylcholine receptor (nAChR) subunit expression was detected by real-time PCR. The production of LDLR was demonstrated by immunofluorescence staining. nAChR-mediated LDLR induction was examined by pre-incubation of the cells with its specific inhibitor, α-bungarotoxin (α-BTX). The functional importance of transcription factor specific protein 1 (Sp1) was examined by luciferase assay, mithramycin pre-incubation or by small interfering RNA (siRNA) transfection. The specific binding of Sp1 to R3 region of LDLR 5’-untranslated region was demonstrated with electrophoretic mobility shift assay (EMSA) and streptavidin-agarose precipitation assay followed by western blotting. The results confirmed that nicotine induced LDLR expression at the transcriptional level. Nicotine was sensed by nAChR and the signal was transduced by Sp1 which bound to the R3 region of LDLR gene. Augmented production of LDLR in the gingival epithelial cells was further demonstrated by immunofluorescence staining using the gingival tissues obtained from the smoking patients.

**Conclusions:**

Taken together, the results suggested that nicotine might contribute to the development of both cardiovascular and periodontal diseases by inducing the LDLR in OECs thereby disturbing lipid metabolism.

## Introduction

Cigarette smoking is an important environmental risk factor for the development of several diseases, including obesity, atherosclerosis, Crohn’s disease, and periodontal disease [1-3]. More than 7,000 different chemicals are found in cigarette smoke. Among them, nicotine has been most extensively investigated, and has been found to have a wide variety of biological effects [4]. For example, nicotine induces the release of catecholamine, thereby raising blood pressure [4]. Nicotine also induces proliferation of vascular smooth muscle cells, which is an important contributor to the growth of atherosclerotic plaques. Moreover, nicotine contributes to the progression of plaque formation by angiogenic activity through the nicotinic acetylcholine receptor (nAChR), and can also disturb lipid metabolism [4,5]. All these effects promote the development of pathological processes contributing to atherosclerosis; however, direct evidence for nicotine’s contribution to atherosclerosis is not currently available. 

Tobacco smokers are known to be at increased risk not only for cardiovascular disease but also for periodontitis [6]. Altered catecholamine release increases the concentration of LDL in the blood and promote atherogenesis [7]. A cohort analysis revealed a correlation between the presence of periodontal pockets and elevated concentrations of total cholesterol and low-density lipoprotein (LDL)-cholesterol [8,9], supporting the increased prevalence of cardiovascular mortality among patients with periodontal disease. Furthermore, in crevicular fluid from periodontitis patients, elevated levels of oxidized LDL (OXLDL) have been detected [10]. In fact, periodontal patients with impaired cholesterol metabolism had deeper periodontal pockets than those with normal metabolic status [11]. OXLDL can bind to scavenger receptors expressed on gingival epithelial cells, and induce the expression of interleukin-8 (IL-8). Thus, secreted IL-8 might augment the inflammatory reaction in gingival tissue [12]. All these reports suggest that abnormalities in lipid metabolism may be involved in the development of periodontitis. Despite these reports, little is known about the relationship between cigarette smoking and cardiovascular and periodontal diseases. Nicotine can readily adsorb to buccal and gingival surfaces [13]. Epithelial cells cover the surface of oral mucosa and are first to be exposed to nicotine following inhalation of cigarette smoke. The effects of nicotine on the cellular functions of gingival epithelial cells have been reported in various experimental settings [14-16]. In a previous report, we comprehensively analyzed the gene expression profile of oral epithelial cells (OECs) after exposure to nicotine by DNA microarrays [17]. The results revealed up-regulated expression of various genes including inflammatory cytokines, signaling molecules, and catalytic enzymes. In the present study, we focused on the expression of the low-density lipoprotein receptor (LDLR) gene. The aim of this study was to evaluate the effect of nicotine on the expression of LDLR gene and to elucidate the signaling pathways leading to nicotine-mediated up-regulation of LDLR. We found that nicotine was able to transcriptionally up-regulate the expression of the LDLR gene in OECs. Our results revealed a possible mechanism by which nicotine-mediated abnormalities in lipid metabolism lead to the development of cardiovascular and periodontal diseases.

## Materials and Methods

### Reagents

Nicotine, nAChR inhibitor α-bungarotoxin (αBtx), and chloride hydrate mithramycin were purchased from Sigma-Aldrich (Tokyo, Japan). 

### Cell culture and Nicotine stimulation

Human oral squamous cell carcinoma (OSCC)-derived cell lines, Ca9-22 and HSC3, were obtained from the Health Science Research Resources Bank (Osaka, Japan). The cells were maintained with RPMI 1640 medium supplemented with 10% FCS, 50 μg/ml streptomycin, and 50 U/ml penicillin (10% FCS-RPMI). Cells were plated on a 24-well cell culture plate (Greiner, Tokyo, Japan) at a density of 2 × 10^5^/well the day before the experiment. The cells were treated with graded concentrations of nicotine for the indicated times. At the end of nicotine stimulation, total RNA was purified using an RNeasy mini kit (QIAGEN, Tokyo, Japan). Oneμg of total RNA was subjected to first-strand cDNA synthesis with Superscript III reverse transcriptase (Life Technologies, Carlsbad, CA), as previously described [18]. Real-time PCR was performed using LightCycler ® Nano (Roche, Tokyo, Japan) with SYBR green (TaKaRa, Tokyo, Japan). The LDLR primers were purchased from TaKaRa. The following primers were used for β-actin. 5ʹ-GGAGCAAGTATCTTGATCTTC-3ʹ (forward), 5ʹ-CCTTCCTGCGCATGGAGTCCTG-3ʹ (reverse). To detect nAChR subunits, the primers listed in Table 1 were used for real-time PCR , as previously described [18]. The primary cultured human gingival epithelial cells were obtained from two patients at Nihon University Dental Hospital (Tokyo, Japan) with the written informed consent (approved by The Ethics committee of Nihon University). The total RNA was isolated and subjected to real-time PCR.

**Table 1:** Primers used for detecion of nAChR subunits.

Gene		Forward Primer	Reverse Primer		GeneBank accession number		
*alpha2*		ACACTTCAGACGTGGTGATTG	CCACTCCTGTTTTAGCCAGAC		U62431		
*alpha3*		TGAGCACCGTCTATTTGAGCG	TGGACACCTCGAAATGGATGAT		U62432		
*alpha5*		ACGTTTTGAAGGGACCAGTACG	ACTCACAATCTCCCATTCTCCAT		U62434		
*alpha7*		GCTGGTCAAGAACTACAATCCC	CTCATCCACGTCCATGATCTG		U40583		
*alpha9*		AAATCTGGCACGATGCCTATC	GCAGGACCACATTGGTGTTCA		AY123244		
*beta1*		CTCTGGACATTAGCGTCGTGG	GCTGAACACCATAGTGCAATTCT		NM_000747		
*beta2*		CAATGCTGACGGCATGTACGA	CACGAACGGAACTTCATGGTG		U62437		
*beta3*		ATCGCCGAAAATGAAGATGCC	GCCACACATTGGTTGTCATCA		U62438		
*beta4*		AACCCGTTACAATAACCTGATCC	ATTCACGCTGATAAGCTGGGC		U62439		

Small interfering RNA (siRNA) transfection - Cells were plated as described above. The siRNA against Sp1 and control siRNA were purchased from Agilent Technologies (Tokyo, Japan). The transfection was performed using RNAi/MAX as described previously [18]. The silencing effect of siRNA was evaluated with real-time PCR.

### Cloning of the 5ʹ-UTR of LDLR gene and luciferase assay

The luciferase construct containing a 271 bp fragment of the LDLR gene promoter spanning from -319 to -64 (+1 corresponds to the A of the ATG translation initiation codon) was amplified using the forward primer 5ʹ-CTGAGCTCCAGCTCTTCACCGGAGACC-3ʹ and the reverse primer: 5ʹ-CTGCTAGCCCTGCTGTGTCCTAGCTGGAAA-3ʹ. The amplified fragment was subcloned into the SacI and Nhe I sites of a pGL4-basic vector (Promega, Tokyo, Japan). This reporter construct was designated wild type (WT). Using the WT plasmid as a template, deletion mutants lacking regions 1, 2, and 3 were made with the QuikChange Site Directed Mutagenesis Kit (Agilent Technologies, Tokyo, Japan). These constructs were designated as R1, R2, and R3, respectively. Ca9-22 cells were seeded into 48-well culture plates (1 × 10^5^ cells/well) and incubated at 37 °C in a humidified atmosphere of 5% CO_2_ for 18 h. Cells were then co-transfected with a luciferase reporter vector (500 ng/well) and the pRL-CMV internal control plasmid (20 ng/well) (Promega) using Lipofectamine (1 μl/well) and Plus Reagent (1 μl/well) (Life Technologies). After 3 h, transfection media was replaced with fresh 10% FCS-RPMI, and further cultured for 3 h. Cells were treated with or without 1 or 10 μM mithramycin (Sigma-Aldrich) for 1 h. After treatment, the cells were stimulated with 100 μM nicotine for the indicated durations. Cell lysates were harvested using passive lysis buffer (65 μl /well). Luciferase activity was measured with a Dual-Luciferase Assay System (Promega), and normalized to the internal control.

### Immunofluorescence staining

Human gingival tissue was obtained during periodontal surgery at the Dental Hospital of Nihon University School of Dentistry, Tokyo, Japan. All patients provided written informed consent and the study protocol was approved by the ethics committee of Nihon University. The properties of all patients were shown in Table 2. The tissues were fixed immediately after excision with 5% acetic acid in ethanol for 18 h. The tissues were embedded in paraffin, and 4 μm specimens were prepared. After deparaffinization, non-specific binding was blocked with 1% BSA/PBS for 1h. The specimens were incubated with a rabbit anti-human LDLR antibody (Ab) (SantaCruz Biotechnology, Santa Cruz, CA) (diluted 100-fold with 1% BSA/PBS) for 18 h. Specimens were washed with PBS 3 times, and further incubated with FITC-conjugated goat anti-rabbit IgG (H+L) Ab (Jackson ImmunoResearch, West Grove, PA) for 2 h. Counterstaining was performed by incubating the specimens with monomeric cyanine nucleic acid stains (Life Technologies) (diluted 500 × with PBS) for 10 min. The staining intensities were evaluated with the following criteria. 0; no-staining, 1; positive only for the superficial keratin layer, 2; positive for keratin and spinal layer, 3; positive for all layers. For Ca9-22 staining, the cells were plated on coverslips, and stimulated with or without nicotine for 12 h. After stimulation, the cells were subjected to immunofluorescence staining as described above.

**Table 2:** Properties of patients.

patient	sex	age	smoking	duration(years)	cigarretes/day	score
1	M	51	Yes	20	10	3
2	M	58	No	0	0	1
3	M	51	Yes	25	15	1
4	M	56	Yes	25	10	2
5	M	46	Yes	26	13	0
6	M	56	Yes	24	10	1
7	M	61	Yes	20	10	ND
8	F	59	Yes	25	10	3
9	F	32	Yes	12	13	3
10	M	52	Yes	30	10	ND
11	M	55	Yes	25	10	ND
12	M	69	Yes	40	20	2
13	M	52	No	0	0	0
14	W	56	No	0	0	0
15	M	50	Yes	30	5	3
16	F	41	No	0	0	1
17	M	27	No	0	0	1

M: male, F: female

ND: not determined

### Electrophoretic mobility shift assay (EMSA)

For EMSA, biotinylated and unlabeled R2 and R3 probes were purchased from FASmac (Shizuoka, Japan). Nuclear extract was purified from nicotine-stimulated or non-stimulated Ca9-22 cells using TransFactor extraction kits (Clontech, Tokyo, Japan). The protein concentrations were measured using a protein assay kit (BioRad). EMSA assays were performed with the EMSA Assay Kit (Affimetrix, Santa Clara, CA). Briefly, 4 μg of nuclear extracts were mixed with 1 μl of poly d(I-C)(1 μg/μl), 2 μl of 5 × binding buffer and 2 μl of nuclease-free water, and incubated for 5 min at room temperature. After incubation, biotinylated probes were added and further incubated for 30 min at 15 °C. For cold inhibition assays, the unlabeled probes were added to the reaction mixture before addition of biotinylated probe. The samples were loaded on native polyacrylamide gel electrophoresis (PAGE) and transferred to a nylon membrane. Western blotting was performed as described previously (18). Streptavidin-HRP was diluted to 1,000 × with 1% BSA-PBST (0.1% tween-20/PBS). For streptavidin-agarose (Stre-Av) precipitation assays, the nuclear extracts from nicotine-stimulated Ca9-22 cells were incubated with biotinylated R2 or R3 probes in the presence or absence of unlabeled R3 probe as explained above. The samples were precipitated by incubating with 10μl of Stre-Av for 18 h at 4 °C. After extensive washing, the samples were loaded on native PAGE and subjected to Western blotting. The primary anti-human LDLR Ab (SantaCruz) and HRP-conjugated goat anti-rabbit IgG Ab (Jackson ImmunoResearch) were diluted to 1000 × and 5,000 ×, respectively, with 1% BSA-PBST. 

### Statistical analysis

**Figure 1:**
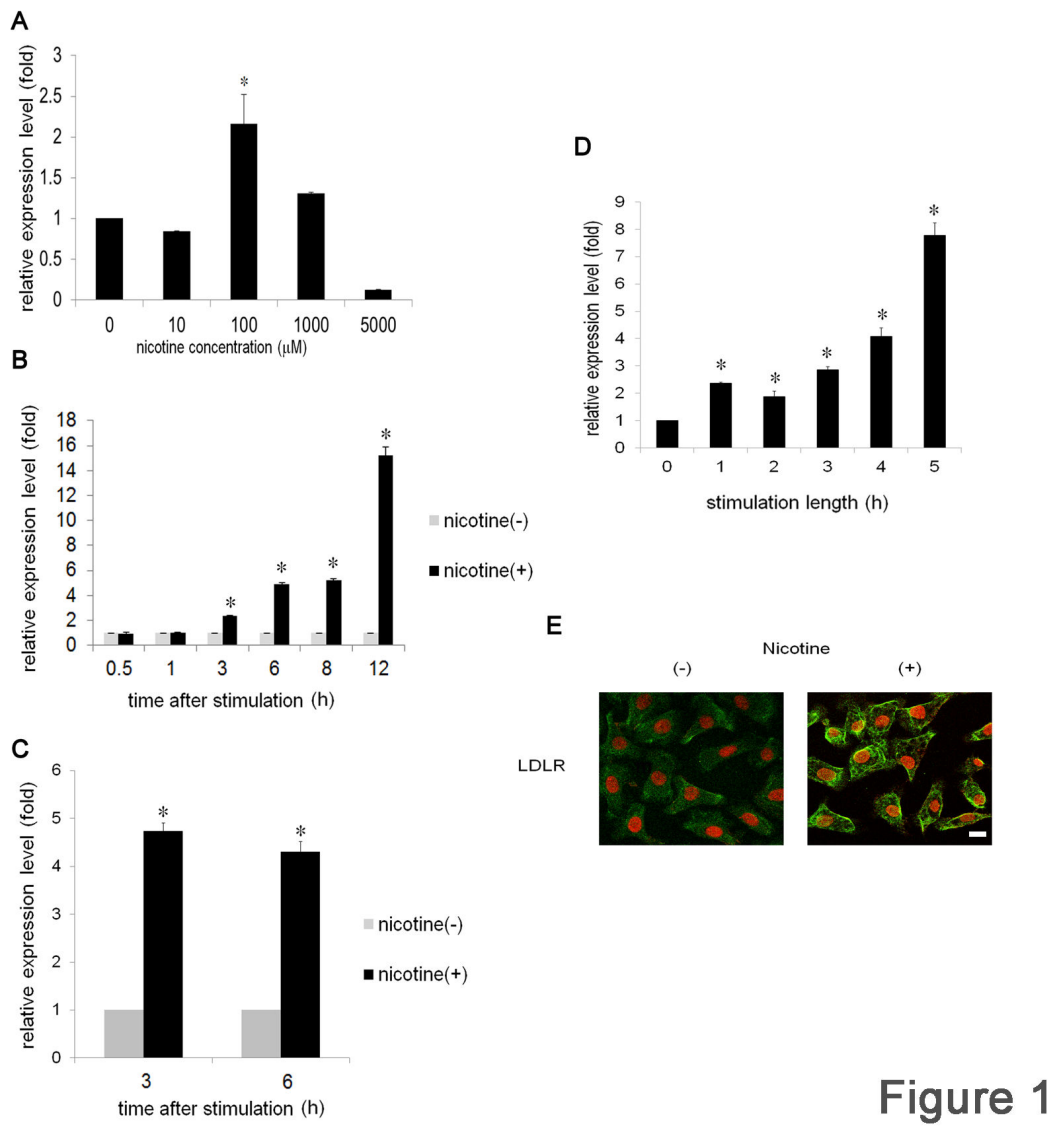
Nicotine induces LDLR expression in OSCC. Ca9-22 cells were stimulated with various concentrations of nicotine for 3 h (A) or with 100 μM of nicotine for various durations (B). (C) HSC3 cells were stimulated with or without 100 μM of nicotine for 3 and 6 h . (D) Ca9-22 cells were stimulated with 100 μM of nicotine for the indicated time. After stimulation, the cells were washed with PBS, and further cultured with fresh medium for 6 h in total. LDLR mRNA expression levels were examined by real-time PCR. Data from at least 3 separate experiments are shown (mean ± SD). *p < 0.05. (E) Ca9-22 cells were stimulated with or without 100 μM of nicotine for 12 h. Localization of LDLR was detected by immunofluorescence staining with anti-LDLR Ab followed by a FITC-conjugated goat anti-rabbit IgG Ab. Green, LDLR; red, nuclei with monomeric cyanine nucleic acid stain. Scale bar (white line): 10 μm.

**Figure 2:**
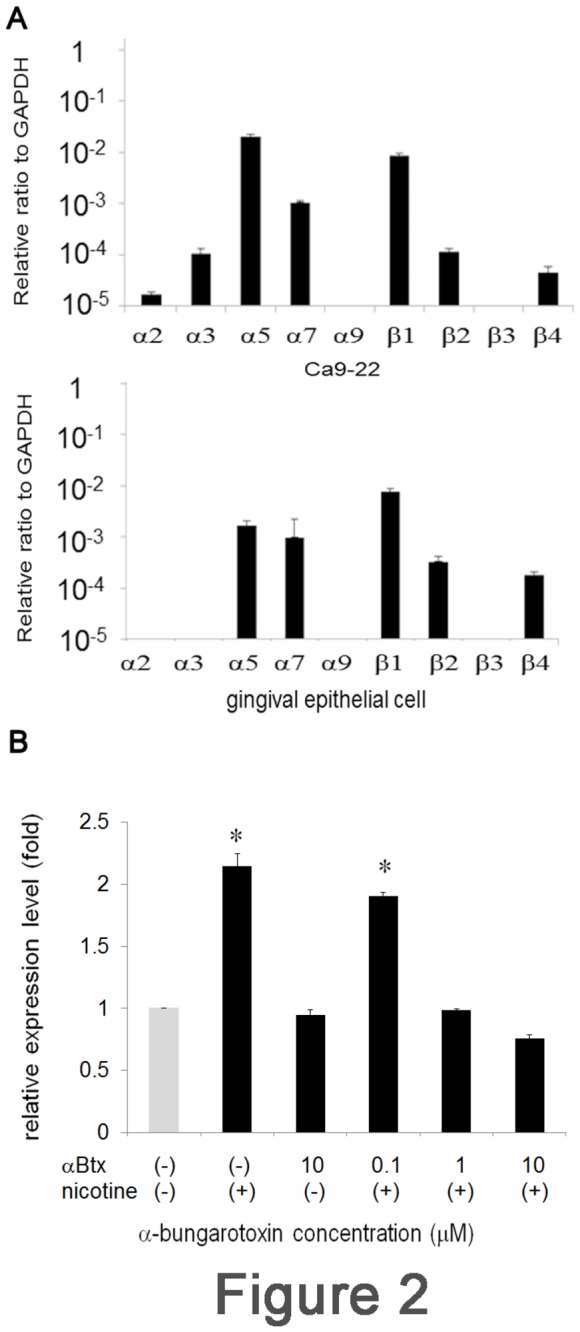
Nicotine-mediated LDLR up-regulation is dependent on nAChR. (A) The expression of nAChR subunits was assessed by real-time PCR for Ca9-22 (upper panel) and primary cultured human gingival epithelial cells (lower panel). The expression level of GAPDH was set as 1. Relative expression level of each nAChR subunit to GAPDH was shown. (B) Ca9-22 cells were pre-treated with graded concentrations of αBtx for 1 h. The cells were washed and further incubated with 100 μM of nicotine for 3 h. LDLR expression was examined by real-time PCR. Data from at least 3 separate experiments are shown (mean ± SD). *p < 0.05.

**Figure 3:**
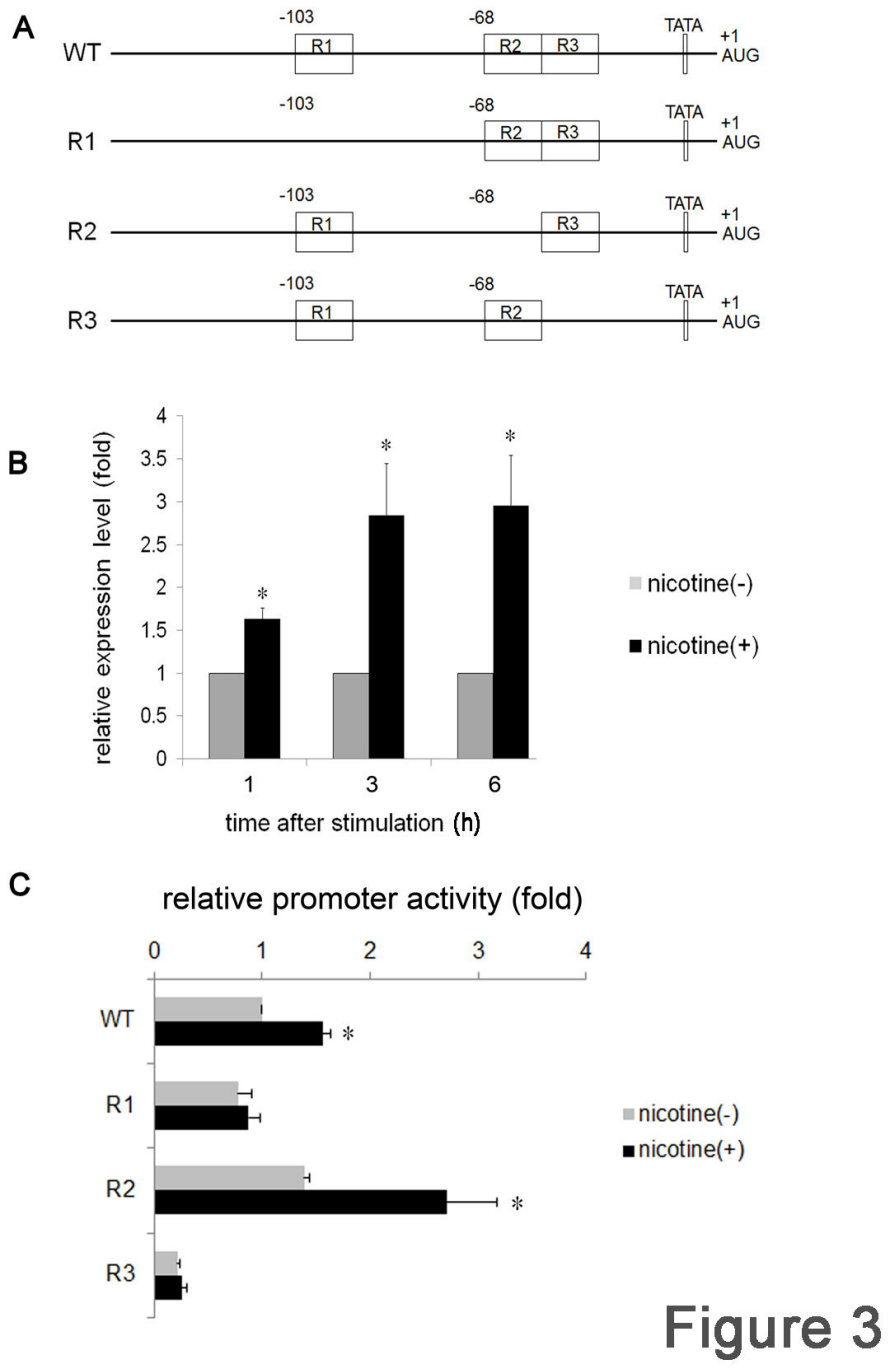
Distinct contributions of R1, R2, and R3 of LDLR gene regulatory regions to nicotine-mediated LDLR expression. (A) Schematic illustration of the 271 bp of the 5ʹ-UTR of the LDLR gene. Nucleotide numbering is relative to the translation initiation site AUG where A is +1. The positions of R1 (-103), R2, and R3 (-68) were indicated as boxes. WT indicates the wild type structure. R1, R2, and R3 represent the mutant constructs lacking each sequence. Each fragment was subcloned in the pGL4-basic vector and used for luciferase assays. Ca9-22 cells were transfected with WT (B) or with R1, R2, or R3 (C) along with normalized pRL-CMV vector for 3 h. After transfection, the cells were stimulated with or without nicotine for 3 h and luciferase activity was measured. At least 3 independent experiments were performed. The data are presented as mean ± SD. *p < 0.05.

**Figure 4:**
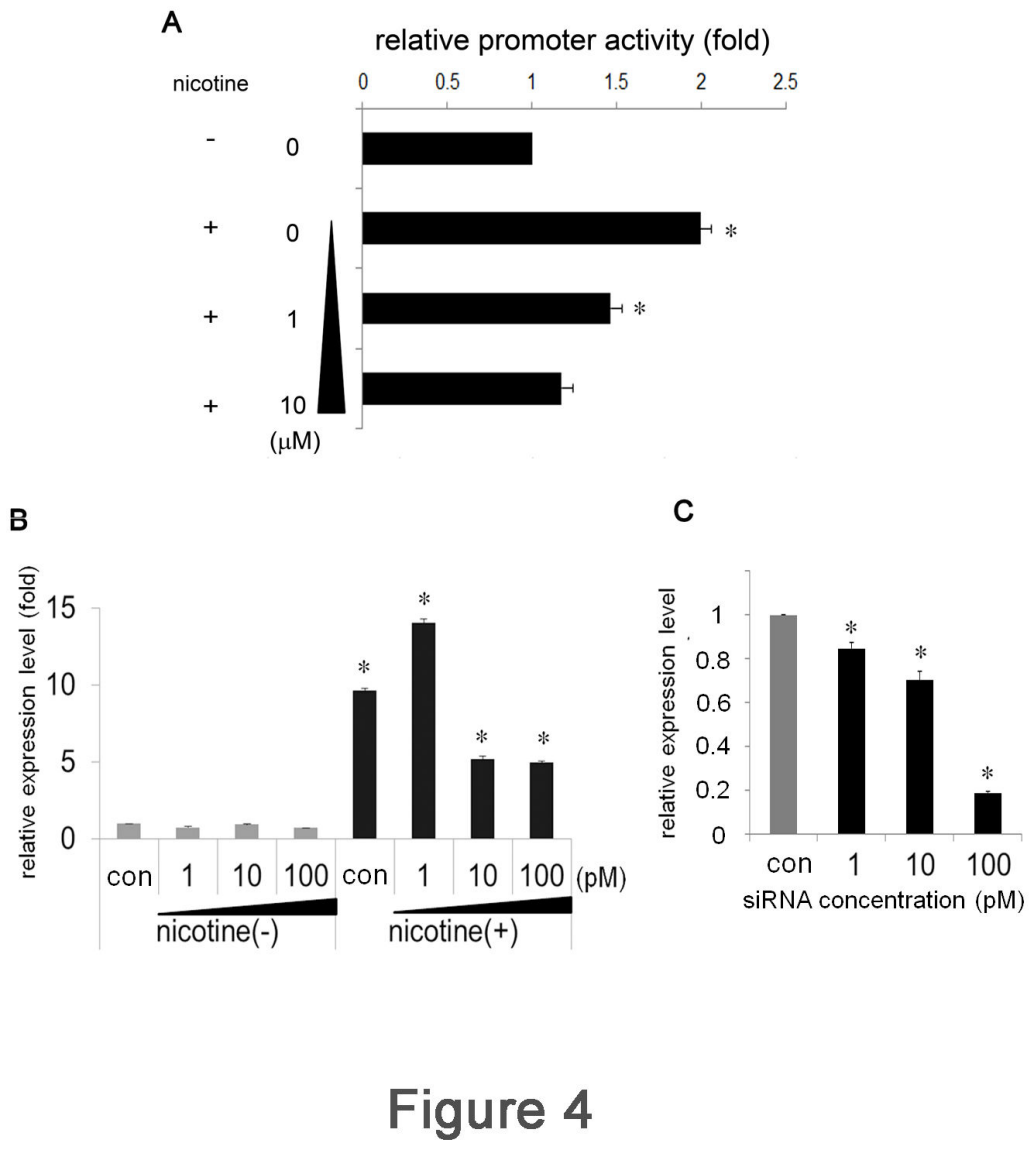
Sp1 plays an important role in nicotine-mediated LDLR expression. (A) Ca9-22 cells were pre-treated with or without mithramycin for 1 h. After washes, the cells were stimulated with 100 μM of nicotine for 3 h and luciferase activity was measured. (B) Ca9-22 cells were transfected with various concentrations of siRNA against Sp1 or control siRNA (con) for 3 h. After transfection, the cells were stimulated with or without 100 μM of nicotine for 3 h. The expression level of LDLR mRNA was examined by real-time PCR. (C) The silencing effect of siRNA transfection on the expression of Sp1 was assessed by real-time PCR. Sp1 expression level of control siRNA-transfected Ca9-22 cell was set as 1. The data are presented as mean ± SD of at least 3 separate experiments. *p < 0.05.

The one-way ANOVA with post-hoc Bonferroni multiple comparison test was used for Figure 1A, B, C, D, Figure 2B, Figure 3B, C, Figure 4A, B and C. Results were presented as mean ± SD values. For staining score, student’s *t*-test was used (Table 2). P values of < 0.05 were considered statistically significant.

## Results

### Nicotine induces expression of LDLR

Comprehensive microarray analysis of a gene expression profile of nicotine-stimulated Ca9-22 cells revealed increased expression of LDLR mRNA [17]. To confirm this result, we first performed real-time PCR. Ca9-22 cells were stimulated with or without various concentrations of nicotine for 3 h. In the absence of nicotine stimulation, low level LDLR expression was observed in Ca9-22 cells and this value was set as 1. A 2.16-fold increase in LDLR expression was detected at 100 μM nicotine (Figure 1A). Expression was decreased at 1,000 μM and no expression was detected at 5,000 μM. Based on these results, we next attempted to determine the time-course of LDLR expression. Cells were stimulated with 100 μM nicotine for various periods of time, as indicated in Figure 1B. Nicotine stimulation gradually increased expression of LDLR mRNA, and, at 3 h and 6 h of stimulation, the level of expression reached to 2.38- and 4.93- fold of non-stimulated cells, respectively (Figure 1B). The expression increased to 15.1-fold at 12 h and returned to the baseline level at 24 h (data not shown). LDLR up-regulation was examined in another cell line, HSC3, and showed a similar pattern of increased expression; however, peak induction was detected after 3 h of stimulation (Figure 1C), and was maintained until 6 h. In the above experiments, the cells were continuously exposed to nicotine. To determine the length of exposure required to induce the expression of LDLR mRNA, Ca9-22 cells were stimulated with nicotine for the indicated times, changed to fresh medium, and further cultured for 6 h in total. The levels of LDLR expression before and after nicotine stimulation were compared. The peak induction was detected at the 5 h point, and expression was increased 7.79-fold (Figure 1D). To further confirm nicotine-induced LDLR up-regulation at the protein level, Ca9-22 cells were stimulated with or without nicotine for 12 h. LDLR expression was examined by immunofluorescence staining. Consistent with the real-time PCR data, low levels of LDLR expression were observed in non-stimulated cells (Figure 1E, left panel). A significant increase was detected in the cells stimulated with nicotine (Figure 1E, right panel). All these results indicated that nicotine up-regulated the expression of LDLR in OSCC.

### nAChR-dependent induction

Numerous studies have revealed the expression and nicotine-sensitivity of nAChR in several OSCC lines [19,20]. The α7-subunit of nAChR plays a pivotal role in transmitting the nicotine signal in OECs [20]. Therefore, we first attempted to examine the expression level of nAChR subunits by real-time PCR. As shown in Figure 2A, the expression level of each subunit varied widely both in Ca9-22 (upper panel) and primary cultured human gingival epithelial cells (lower panel). The results indicated that both cells are capable of transducing the nicotine-binding signal through the nAChR α7-subunit. To reveal the contribution of nAChR in nicotine-mediated LDLR expression, cells were pre-incubated with graded concentrations of the nAChR-specific inhibitor, αBtx, for 1 h, and stimulated with nicotine. After stimulation, the expression of LDLR was measured by real-time PCR. LDLR mRNA was successfully induced by nicotine stimulation (Figure 2B). Pre-incubation of the cells with αBtx reduced the expression of LDLR mRNA in a dose-dependent manner (Figure 2B). At a concentration of 1 μM αBtx, LDLR expression returned to the baseline level. Incubation with αBtx alone did not affect the LDLR expression level, indicating specific inhibition (Figure 2B). These results indicated that nAChR contributed to the increased expression of LDLR mRNA.

### Transcriptional regulation of LDLR expression

LDLR up-regulation was detected as an increase in mRNA expression, and would expectedly be controlled at the transcriptional level. To clarify the underlying signaling mechanisms, we next performed luciferase assays. The 271 bp 5ʹ-UTR of the LDLR gene was cloned by PCR and inserted into the luciferase reporter plasmid, pGL4-basic vector. The structure of this region was schematically illustrated in Figure 3A. Ca9-22 cells were transfected with this plasmid along with a normalized vector, and luciferase activity was measured after the indicated times of stimulation. Luciferase activity was increased by nicotine, and reached to 2.84-fold after 3 h of stimulation (Figure 3B). Increased activity was maintained until 6 h. These results indicated that nicotine stimulation could lead to transcriptional activation of the LDLR gene in Ca9-22 cells.

As illustrated, this 271 bp region contains 3 imperfect direct repeat sequences, R1, R2 and R3, and a putative TATA-like sequence [21] (Figure 3A). These sequences are each 16 bp in length, and are located at nucleotide position -103 and -68 with respect to the major transcription initiation site (R2 and R3 are adjacent to each other). R3 has a consensus binding sequence for the transcription factor, Spl, and plays a pivotal role in LDLR transcription [21,22]. To examine the contribution of these repeats, we constructed 3 deletion mutants, with each mutant lacking R1, R2, or R3 (Figure 3A), and each construct was subjected to luciferase assay. Consistent with the results reported above, nicotine increased luciferase activity to 1.56-fold at 3 h in the WT. In the R1 mutant, luciferase activity was equivalent to WT in the absence of nicotine. Interestingly, nicotine did not increase luciferase activity in the R1 mutant. In contrast, baseline luciferase activity was slightly increased in the R2 mutant compared with that of WT, but was further increased by nicotine stimulation (2.71-fold). In contrast, luciferase activity was drastically reduced in the R3 mutant, and nicotine stimulation did not influence luciferase activity. Collectively, these results indicated that each repeat plays a distinct role in nicotine-mediated LDLR transcription, and suggested a contribution of Sp1 to LDLR regulation.

### Contribution of Sp1 to nicotine-mediated LDLR expression

To further examine the contribution of Sp1 to LDLR expression, WT transfectants were pre-incubated with graded concentrations of the Sp1-specific inhibitor mithramycin [23] for 30 min, and then stimulated with 100 μM nicotine for 3 h. Consistent with the above results, nicotine increased luciferase activity by 2.00-fold after 3 h in the absence of mithramycin. In contrast, luciferase activity was reduced by mithramycin treatment in a dose-dependent manner. Half-maximum inhibition (IC 50) was observed at a 1 μM of mithramycin (Figure 4A). We next performed RNA silencing experiments to confirm the necessary role of Sp1 in nicotine-mediated LDLR expression. Ca9-22 cells were transfected with siRNA against Sp1 and control siRNA (con). The transfectants were stimulated with or without nicotine for 3 h, and the expression of LDLR was measured by real-time PCR. Sp1 siRNA transfection did not affect the baseline expression of LDLR mRNA (data not shown). Stimulation of control siRNA-transfected cells (con) with nicotine up-regulated luciferase activity 9.63-fold (Figure 4B). In contrast, luciferase activity was reduced in Sp1 siRNA-transfected cells in a concentration-dependent manner. At 100 pM of siRNA, luciferase activity was reduced to half that of control siRNA-transfected cells. The silencing effect of siRNA on the expression of Sp1 was assessed by real-time PCR (Figure 4C). These results further confirmed the importance of Sp1 in nicotine-mediated LDLR expression.

### Detection of Sp1 binding to R3 by EMSA

**Figure 5:**
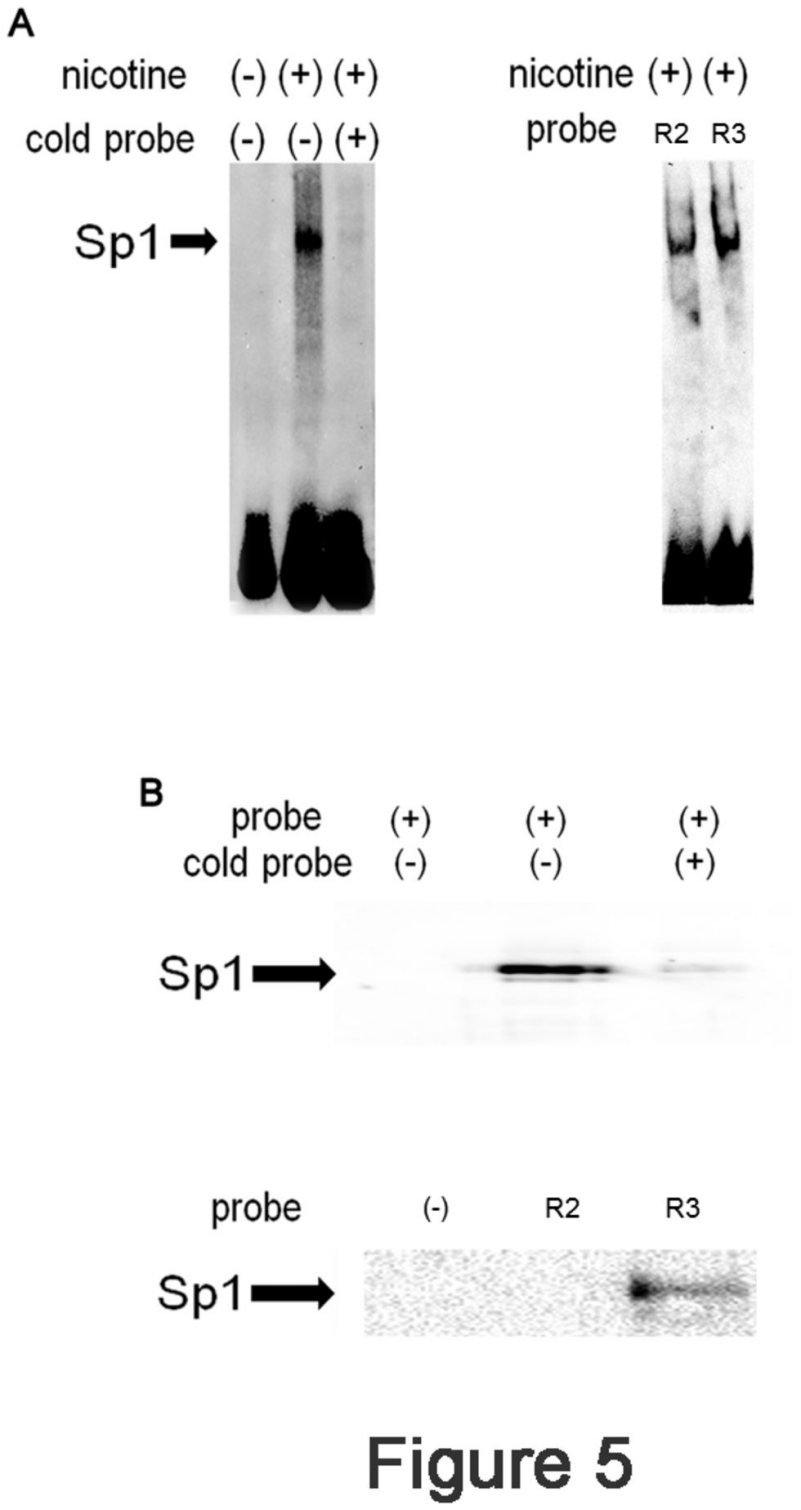
Nicotine stimulation leads to Sp1 binding to R3. (A) Left panel: Ca9-22 cells were stimulated with (+) or without (-) nicotine for 3 h. After stimulation, the cells were harvested and the nuclear extracts were prepared. Twenty μg of nuclear extract was incubated with biotinylated R3 probe with (+) or without (-) cold R3 probe. The samples were separated by native PAGE and transferred to a nylon membrane. The retarded band was detected by incubating the membrane with streptavidine-HRP followed by ECL. The representative of 3 separate experiments was shown. Right panel: Nicotine-stimulated nuclear extract was incubated with or without R2 or R3 probe and the retarded bands were detected as described above. (B) Nicotine-stimulated nuclear extracts were prepared from Ca9-22 cells and subjected to Stre-Av precipitation assays. Upper panel: lane 1: Stre-Av precipitation assay performed without nuclear extract. Lane 2: Stre-Av precipitation assay with nuclear extract. Lane 3: Stre-Av precipitation assay with biotinylated and non-labeled R3 probes. Lower panel: lane 1: Stre-Av precipitation assay performed without probe. Lane 2: Stre-Av precipitation assay with R2 probe. Lane 3: Stre-Av precipitation assay with R3 probe.

The above results suggested the fundamental role of Sp1 in nicotine-mediated expression of LDLR. To further explore the importance of Sp1, the binding of Sp1 to R3 was examined by EMSA. Nuclear extracts were prepared from nicotine stimulated or non-stimulated Ca9-22 cells. A biotinylated R3 probe was incubated with the nuclear extracts. The samples were separated by native PAGE, and interactions were detected by a streptavidin-HRP conjugate, followed by a chemiluminescence reaction. As shown in Figure 5A (left panel), a retarded band was detected in nicotine-stimulated (lane 2), but not in unstimulated (lane 1) nuclear extract. When the non-biotinylated cold probe was added to the reaction mixture, the retarded band completely disappeared (lane 3). Furthermore, EMSA assay was performed with R2 probe (Figure 5A, right panel) and the retarded band was also detected. To further confirm the R2-Sp1 or R3-Sp1 interaction, we performed a Stre-Av precipitation assay. The biotinylated R3 probe was incubated with or without niocotine-stimulated nuclear extracts. The samples were incubated with Stre-Av and subjected to western blotting. As shown in Figure 5B (upper panel), Sp1 was detected only when the nuclear extract was incubated with biotinylated R3 probe (lane 2). Addition of the cold probe to the reaction diminished the Sp1 band (lane 3), indicating the specificity of the reaction. In contrast, Sp1 was not detected in R2 probe (Figure 5B, lower panel, lane 2). All these results indicated that nicotine stimulation leads to the binding of Sp1 to R3.

### LDLR expression in gingival epithelial cells

**Figure 6:**
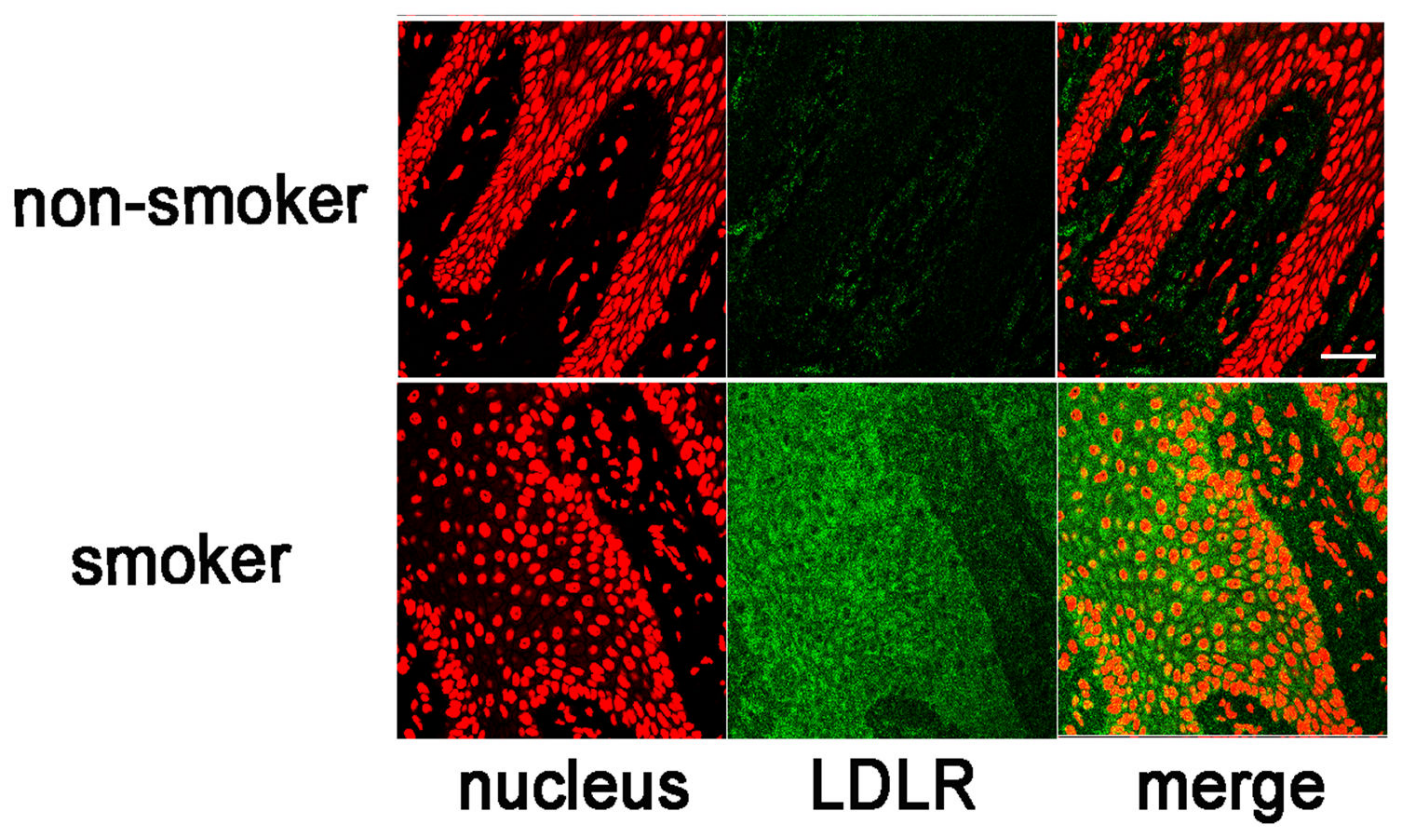
Increased expression of LDLR in the gingival epithelium from smoking patients. (A) The gingival tissues were excised from smoking (lower panel) or non-smoking patients (upper panel) and fixed with 5% acetic acid-ethanol. The tissues were embedded in paraffin and 4 μm sections were prepared. The specimens were incubated with anti-LDLR Ab followed by FITC-conjugated goat anti-rabbit IgG Ab. Images were viewed and photographed using a LSM510 confocal laser microscope (Carl Zeiss, Heidelberg, Germany). Green, LDLR; red, nuclei with monomeric cyanine nucleic acid stain. Scale bar (white line): 50 μm.

If nicotine increases LDLR expression in OECs, tobacco smoking should increase LDLR expression. We therefore examined whether increased expression of LDLR was observed in the gingival epithelial cells of tobacco smokers. Gingival tissues were excised from patients with informed consent and subjected to immunofluorescence staining. The properties of all patients were shown in Table 2. The age of the patients ranged from 27 to 69 years, with the mean age of 51.2 years. The patients include 13 men and 4 women, at a ratio of 3.25:1. The duration and number of cigarette usage per day was shown. In the gingival epithelium of non-smokers, LDLR expression was mainly detected in the keratin layer, with weak expression also observed in the spinal layer (Figure 6A, upper panel). In contrast, LDLR expression was drastically increased in gingival tissue from smoking patients. LDLR expression was observed throughout the epithelial layers (Figure 6A, lower panel), and staining intensities were found to be much stronger in the epithelium obtained from smokers. According to the criteria described in Materials and methods, the staining intensities were evaluated and scored (Table 2). The mean scores for non-smokers and smokers were 0.6 vs 2.0 and the difference was statistically significant (p=0.023). ND means that the score could not be determined because of the small epithelial cell region. These results indicated that smoking leads to the increased expression of LDLR in gingival epithelial cells.

## Discussion

In the present study, exposure of OECs to nicotine was demonstrated to induce the expression of LDLR at the transcriptional level. To the best of our knowledge, this is the first report indicating the direct relationship between nicotine and LDLR in OECs.

Although LDLR induction was observed in both Ca9-22 and HSC3 cell lines, the responsiveness of the cell lines differs slightly. In Ca9-22, the expression of LDLR mRNA was augmented time-dependently and peaked at 6 h after stimulation. On the other hand, in HSC3, the peak induction was observed at 3 h. These differences may be due to basic cellular activity levels. In fact, both cell lines secrete interleukin-8 spontaneously and the concentration is higher in HSC3 cells. To determine the optimal stimulation conditions, Ca9-22 was exposed to nicotine for various lengths of time, and 5 h of continuous stimulation was found to be the most effective for inducing expression of LDLR mRNA. We used 100 μM of nicotine to stimulate the cells. Although this concentration corresponds to the nicotine concentration of the plasma of inhalating smokers [13], we still do not know whether levels in the oral cavity is equivalent to this condition. It should be necessary to measure the actual nicotine concentration in the oral cavity right after smoking. 

Nicotine can be absorbed into the body through several different routes, in a pH-dependent manner. The pH of alveoli is around 7.4 and this pH is appropriate for nicotine to pass through the cell membrane and enter the circulation. The concentration of nicotine in the plasma is 30-40 ng/ml and 2.5-8.0 ng/ml in inhaling and non-inhaling smokers, respectively [13]. In our study, Ca9-22 cells responded to 100 μM of nicotine and a concentration approximately 5- to 60- fold higher than the above concentrations. However, the nicotine concentration in the oral cavity reaches a millimolar order immediately after smoke inhalation, suggesting that this concentration range should reflect the actual environment of the oral cavity [13]. Consistent with mRNA induction, immunofluorescence staining experiments showed the apparent up-regulation of LDLR after nicotine stimulation. Interestingly, detection of LDLR on the cell surface of cultured cells was only successful when using fixation with ethanol containing 5 % acetic acid. Moreover, this was the case even in tissue staining; this may be valuable for the further detection of LDLR in several other organs or tissues.

Many reports have indicated that nicotine exerts its biological functions through nAChR. As nicotine can pass through the biological membrane very easily, Dunckley et al. attempted to discriminate between nAChR-dependent and -independent signaling pathways [24]. Their results indicated the importance of nAChR; however, they also observed nAChR-independent signaling. nAChRs are pentameric ligand-gated cation channels composed of 5 subunits and are permeable to Na^+^, K^+^, and Ca^2+^ [5]. The subunit composition of nAChR is important as it affects its Ca^2+^ permeability [25] In gingival epithelial cells, the expression of α3, α5, α7, α9, α2, and β4 subunits of this channel have been reported [26-28]; these subunits transduce the nicotine-binding signal [14,16,20,29]. Consistently, nicotine-mediated effects were inhibited by pre-incubating the cells with the nAChR inhibitor αBtx in our experiment. αBtx is an antagonist for the α7-subunit of nAChR and inhibits its downstream signaling. nAChR signaling is mediated by several different pathways [19,20]. However, to date, there has been no report on LDLR up-regulation via nAChR signaling. 

By removing the cholesterol-carrying LDL from plasma by receptor-mediated endocytosis [30], the LDLR plays a key role in determining plasma cholesterol levels. In this context, regulation of LDLR expression is of particular importance for the maintenance of cholesterol level in the plasma. LDLR regulation has been intensely investigated in terms of sterol concentration in the cytoplasm [31]. The 5′-UTR of LDLR contains R1, R2, and R3 [21,22]. Nicotine failed to induce the luciferase activity in R1 deletion mutant. In contrast to R3 mutant, however, R1 did not alter the baseline transcriptional activity. These results are consistent with the fact that R1 mutation decreased transcription to a slightly lesser extent than R2 or R3 [21,32,33]. R2 contains the sterol regulatory element-1 (SRE-1), which is the binding site for the transcription factor sterol regulatory element binding proteins (SREBPs) of the basic-helix-loop-helix-leucine zipper family [34]. The transcription of LDLR is primarily under the control of the SREBPs. 

SREBPs bind to SRE-1 only when the cells recognize a reduced sterol level. In our experiment, the sterol level was maintained constantly. The baseline luciferase activity with the R2-deletion construct was slightly increased and was further augmented by nicotine stimulation. These results indicated that SRE-1 in R2 is only functional under sterol-deprived conditions; it did not respond to nicotine. Interestingly, we detected the retarded band by EMSA with R2 probe. As this band did not react with anti Sp1 Ab, the retarded band should contain some factor other than Sp1. Further study should be needed to elucidate the nature of this band. In contrast, the luciferase activity was completely abolished with the R3-deletion construct, indicating that R3 is the site fundamental to the maintenance of cellular cholesterol concentration. The importance of R3 for the expression of LDLR is demonstrated in familial hypercholesterolemia, an autosomal dominant disorder of cholesterol metabolism [32,33,35]. The nucleotide substitution of C to G at -139 in the R3 region resulted in the reduction of LDLR mRNA transcription. In the present report, R3 was demonstrated to bind directly to Sp1 in response to nicotine stimulation. Mithramycin treatment and the transfection of siRNA against Sp1 reduced LDLR promoter activity and mRNA expression level, respectively. These results reinforced the importance of Sp1; however, the signaling pathway linking nicotine, nAChR, and Sp1 is obscure. How does nicotine stimulate Sp1 activity? Sp1 has a wide variety of target genes [36]. To induce its target expression, Sp1 has to be activated by post-translational modifications [36,37] and be translocated to the nucleus [38,39]. The nuclear transport mechanism of Sp1 is not well understood; however, Sp1 interacts with importin α through its zinc-finger domain [39]. Intriguingly, several nAChR subunits themselves are the regulatory targets of Sp1 [40-45], suggesting possible feedback control mechanisms. Sp1 interacts with several different proteins [46], for instance, with transcription factors, such as c-Jun [47], Sox10 [48], and NF-Y [49]. Moreover, nicotine enhances the proliferation of lung cancer cells through nAChR. To exert its effect, nicotine induces the expression of peroxisome proliferator-activated receptor β/δ and conversely inhibits the expression of activator protein 2 (AP2) in vitro. Interestingly, the reduction of AP2 is regulated by Sp1 [50]. This is the first report to show the direct relationship between nicotine and Sp1; however, Sp1 activation mechanisms should be addressed by further experiments. 

The fundamental role of LDLR is to take up LDL particles into cells through coated pits by receptor-mediated internalization. LDL is then delivered to the lysosome and is degraded to generate cholesterol. The cholesterol so produced can suppress further generation of LDL-derived cholesterol. Cholesterol is an essential component of the plasma membrane, and intracellular cholesterol levels should be strictly maintained [51]. Essentially, augmented expression of LDLR leads to increased uptake of LDL from blood and reduces the risk of atherosclerosis. In our experiment, nicotine augmented the expression of LDLR in OSCCs. This raises the question of whether nicotine can reduce the risk for atherosclerosis? Contradicting this assumption are the following facts: (1) In LDLR-knockout mice, nicotine increased the size of the aortic lesion [52]. (2) Chronic administration of nicotine to New Zealand white rabbits was found to elevate triglycerides, total cholesterol, and LDL cholesterol [53]. (3) Nicotine administration significantly disturbs the lipid metabolism in rats [54,55]. All these reports suggested the harmful effect of nicotine on atherosclerosis development. 

The initial step in the development of atherosclerosis is the deposition of LDL in the vessel intima. The deposited LDL is oxidized locally and is absorbed by its specific receptor expressed on the surface of macrophages [56], which generate the lesion. In this context, the induction of the OXLDL receptor by nicotine should be of great importance. In fact, the concentration of OXLDL in the gingival crevice is elevated by nicotine inhalation [10]. In analogy to the genesis of atherosclerotic regions, the locally accumulated LDL should be oxidized in the gingival crevice. From this point of view, it could be speculated that the unregulated expression of LDLR in gingival epithelial cells may take up the excess amount of LDL and result in epithelial cell death. LDL emerging from the dead cells would accumulate in the gingival crevice. Thus, accumulated LDL may be oxidized locally and it may result in the further uptake by macrophages and so contribute to the development of atherosclerosis. The biological sequences connecting the nicotine-mediated upregulation of LDLR in OECs and development of atherosclerosis should be clarified in future studies.
